# Is stress related to the presence and persistence of oncogenic human papillomavirus infection in young women?

**DOI:** 10.1186/s12885-021-08010-4

**Published:** 2021-04-16

**Authors:** Ulrike Kuebler, Susanne Fischer, Laura Mernone, Christian Breymann, Elvira Abbruzzese, Ulrike Ehlert

**Affiliations:** 1 Department of Clinical Psychology and Psychotherapy, University of Zurich, Binzmühlestrasse 14/26, 8050 Zurich, Switzerland; 2 University Hospital Zurich, Zurich, Switzerland

**Keywords:** Oncogenic human papillomavirus, Cervical cancer, Chronic stress, Salivary cortisol

## Abstract

**Background:**

Persistent infection with high-risk human papillomavirus (HR-HPV) is the most important risk factor for the development of cervical cancer, but factors contributing to HR-HPV persistence are incompletely understood. The objective of this study was to test for associations of chronic stress and two aspects of diurnal cortisol secretion (i.e., the cortisol awakening response [CAR] and total cortisol output over the day [AUCgday]) with HR-HPV status at baseline and 12 months later (follow-up).

**Methods:**

We evaluated 188 women (25 ± 3 years) at baseline. Follow-up investigation was restricted to HR-HPV infected women at baseline. Of the initial 48 HR-HPV positive participants, 42 completed the follow-up (16 HR-HPV positive and 26 HR-HPV negative). At baseline and follow-up, we determined HR-HPV status in cervical smears, assessed chronic stress, and repeatedly measured salivary cortisol over the day. At baseline, we analyzed salivary cortisol only in a subgroup of 90 participants (45 HR-HPV negative and 45 HR-HPV positive).

**Results:**

At baseline, higher chronic stress (excessive demands at work: *p* = .022, chronic worrying: *p* = .032), and a higher CAR (*p* = .014) were related to baseline HR-HPV positivity. At follow-up, there was a statistical trend for a positive association between the CAR and HR-HPV positivity (*p* = .062). Neither the CAR nor the AUCgday mediated the associations between chronic stress and HR-HPV status.

**Conclusions:**

Our findings suggest that both chronic stress and diurnal cortisol are related to the presence of HR-HPV infection and may thus play a role in HPV-associated cervical carcinogenesis.

## Background

Cervical cancer is the fourth most common cancer in women worldwide, with an estimated incidence of 500,000 cases per year [1, 2]. The most important risk factor for the development of cervical cancer is high-risk human papillomavirus (HR-HPV) infection of cervical mucosa cells [3]. More than 60% of HR-HPV infections are cleared within 6–12 months and cause no pathological changes, but in some women infections persist and can progress to cervical intraepithelial neoplasia (CIN) and cervical cancer [4, 5]. Although the factors and precise mechanisms regulating the course of HR-HPV infection are not completely understood, clinical and epidemiological data revealed an association between HR-HPV clearance and effector functions of T lymphocytes, also referred to as cell-mediated immunity (CMI) [6, 7].

Animal and human studies provide converging evidence that chronic stress compromises CMI, particularly cytotoxic T lymphocyte (CTL) and CD4+ T cell activity, CD4+ T cell and CTL counts, and lymphocyte migration and proliferation [8–15]. There is evidence that the stress effects on the immune system are mediated by glucocorticoids (GCs) [9, 16, 17], which are released from the adrenal cortex into plasma as the end product of the hypothalamus-pituitary-adrenal (HPA) axis in response to stress and thus in characteristic stress-related patterns [18]. A large body of literature indicates prolonged hypersecretion of GCs and heightened GC secretion occurring during the first hour after awakening (cortisol awakening response, CAR) under chronic job and general life stress conditions [19, 20]. Increased GC levels in turn have been shown in vitro and ex vivo to compromise the equilibrium between type 1 T-helper (Th1) cell and Th2 cell activity by differential modulation of cytokine expression, resulting in a shift from a Th1 to a Th2 response [21]. Notably, whereas Th1 cell activity contributes to CMI, Th2 cell activity favors the humoral immune response [22–24]. Hence, increased stress-induced GC levels are presumed to systematically suppress Th1-mediated CMI, while causing a shift toward Th2-mediated humoral immunity.

Considering the suppressive effects of chronic stress and increased GC levels on CMI, and given the importance of cell-mediated immune responses in HR-HPV clearance, chronic psychological stress may participate in promoting HR-HPV persistence and thus HPV-associated cervical cancer progression mediated by cortisol. Indeed, several studies have demonstrated associations between psychosocial stress and prevalence and progression of HPV-associated cervical dysplasia and cancer [25–31], although not unequivocally so [32, 33]. Among the hitherto published studies, however, most used cross sectional or retrospective designs and focused on the association between stress and cervical dysplasia or cancer; only two studies were of prospective nature [31, 32] and only one directly focused on the association between chronic stress (i.e., bereavement) and HR-HPV infection [25].

Concerning mechanisms that might underlie the possible link between psychosocial stress and HR-HPV persistence, a case-control study in Caucasian women provided first evidence for stress-related decrements in proliferative T-cell responses to HPV-16 suggesting suppressed cell-mediated immune responses as mediator [34]. However, to the best of our knowledge, no study has previously examined associations between cortisol and HR-HPV presence or persistence as an additional psycho-endocrine mechanism linking stress and HPV-mediated cervical neoplasia and cancer.

Therefore, the purpose of this study was to test for associations of chronic stress and two aspects of diurnal cortisol secretion (i.e., the cortisol awakening response and total cortisol output over the day), with HR-HPV presence and persistence. We measured chronic stress, salivary cortisol, and HR-HPV status at baseline and 12-months after baseline. Our main hypotheses were that a) higher levels of chronic stress, a higher cortisol awakening response, and a higher total cortisol output would be associated with an increased risk of HR-HPV positivity at baseline (i.e., HR-HPV presence) and 12 months after baseline (i.e., HR-HPV persistence) and that b) the hypothesized association between chronic stress and HR-HPV presence/persistence would be statistically mediated by a higher cortisol awakening response and by a higher total cortisol output.

## Methods

### Participants

This study was part of a large research project, in which a random sample of 188 young women was recruited by aid of collaborating physicians and advertisements. Participants with any self-reported acute or chronic immune-related disease (e.g., autoimmune disorder, HIV-positive status, current infectious diseases) were not eligible for the study. Explicit exclusion criteria, obtained by participants’ self-report, were: age < 18 or > 31 years, virginity, HPV vaccination, pregnancy in the past or currently pregnant, irregular gynecological check-ups (i.e., more than 2 years in between visits, representing the standard in Switzerland), HPV diagnosis at last gynecological check-up, treatment of cervical abnormalities during the last year, and previous cervical conization.

**Fig. 1:**
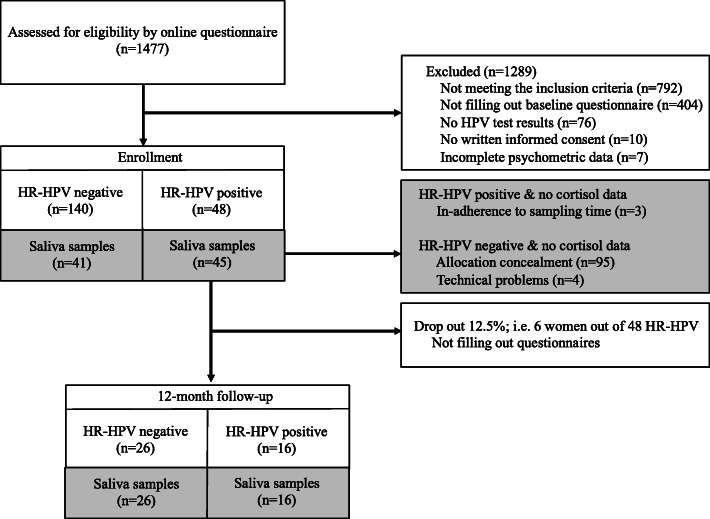
Participant flow throughout the study

Of the total of 1477 participants assessed for study eligibility, 188 participants were enrolled (see Fig. 1 for details on participant flow). Of these, 48 participants were tested HR-HPV positive and 140 HPV negative. Based on a power analysis (see statistical analysis) and for reasons of limited funding, we analyzed salivary cortisol in 90 participants (i.e., 45 HR-HPV negative [cortisol-control group] and 45 HR-HPV positive). Notably, three participants of the HR-HPV positive group had to be excluded because of non-adherence to sampling time (see study procedure). The allocation of HR-HPV negative participants to the ‘cortisol-control group’ (*n* = 45) was randomly achieved by using 140 opaque and sealed envelopes for all of our 140 HR-HPV negative participants, i.e., one envelope for each HR-HPV negative participant. An independent research assistant generated the random allocation sequence by sealing, mixing, and subsequently opening 45 opaque envelopes. Due to technical problems with assaying, data of cortisol were missing in 4 participants rendering a final sample of 41 HR-HPV negative women and 45 HR-HPV positive women for cortisol measurements.

Follow-up investigation was restricted to women with HR-HPV infection at baseline. Of the initial 48 HR-HPV positive participants, 42 (87.5%) completed the 12-month follow-up. Of these, 16 participants (38%) were still HR-HPV positive while 26 participants (62%) were negative to HR-HPV.

### Procedures

All participants who passed the screening were given detailed written information about the study purpose and procedure. Two weeks before their next routine check-up with their gynecologist, the participants received a link for a baseline online questionnaire (T1), which included questions addressing demographics, chronic stress, and suspected covariates (see self-reported measures for more information). The questionnaire had to be filled in before the routine gynecological check-up. In addition, participants received material (DNAPap™ Cervical Sampler; Qiagen Gaithersburg, Inc., USA) and written instructions for HPV testing by means of a cervical swab, which was to take place during the routine check-up at their gynecologist’s practice. In detail, participants were asked not to schedule their routine gynecological check-up during menstruation, to abstain from vaginal cream, spermicides, and lubricants three days before the examination, and to abstain from sexual intercourse two days before the examination. Cervical samples were collected by healthcare professionals during the routine gynecological check-up. After cervical cells were collected via the DNAPap™ Cervical Sampler, the system was sent to a specialized laboratory for HPV testing (Laboratory Enders & Partners; Stuttgart, Germany).

Moreover, participants received material and written instructions for saliva collection to assess the diurnal cortisol secretion using Salivette collection devices (Sarstedt, Sevelen, Switzerland). All participants were instructed to collect 7 saliva samples over the course of a regular workday within two weeks after HPV testing. For cortisol awakening response assessment, saliva samples were collected immediately after awakening (while still lying in bed), 30, and 60 min thereafter. To assess the cortisol output over the day, samples were collected at 11:00, 14:00, 17:00, and 20:00 h. Participants were asked to abstain from strenuous physical activity and from alcohol consumption 24 h before saliva collection. In addition, participants were asked to abstain from smoking starting on the morning of saliva collection as well as from brushing their teeth, eating and drinking for 20 min prior to sample collection. To control the sampling time self-reports of the exact sampling time were used. Three participants of the HR-HPV positive group had to be excluded from the analysis due to sampling time. Saliva samples were stored in a fridge until sampling was completed and were then sent to the biochemical laboratory at the Institute of Psychology of the University of Zurich, Switzerland, and stored at − 20 C until biochemical analysis.

Follow-up investigation was restricted to women with HR-HPV infection at baseline. For follow-up 12 months after baseline (T2), participants with HR-HPV positive status at baseline were contacted again 1 month before T2. Notably, we chose a follow-up of 12 months because the risk of CIN grade 2 or worse diagnosis by 30 months has been found to be over 50% among women younger than 30 years with HR-HPV infections that persisted at least 12 months [4]. Study procedure as well as psychometric and biochemical measures were analogous to T1. In brief: Participants had to fill in an online questionnaire within a period of 2 weeks before a cervical swab. In addition, they were instructed to collect 7 saliva samples over the course of a regular workday within a period of two weeks after cervical swab.

The Ethics Committee of the University of Zurich, Switzerland, formally approved the research protocol, which was conducted in accordance with the principles of the Declaration of Helsinki. All participants provided written informed consent before participating in the study and were compensated with 120 CHF for their participation.

### Biological measures

#### Assessment of HPV status

Cervical swab cells collected with the DNAPap™ Cervical Sampler were tested for HR-HPV infections at the laboratory Enders & Partners (Stuttgart, Germany) using the hc2 High-Risk HPV DNA Test® from QIAGEN (Gaithersburg, Inc., USA). The hc2 is an in vitro nucleic acid hybridization assay with signal amplifications using microplate chemiluminescence for the qualitative detection of DNA in cervical specimens of 13 of the most frequently found HR-HPV types in cervical tumors [35], i.e., HR-HPV 16, 18, 31, 33, 35, 39, 45, 51, 52, 56, 58, 59, 68. The assay was performed with cutoff of 1.0 pg/ml recommended by Food and Drug Administration (FDA).

#### Cortisol analysis

At baseline, we analyzed salivary cortisol only in a subgroup of 90 participants (i.e., 45 HR-HPV negative and 45 HR-HPV positive) due to reasons of limited funding (see section study participants). Thawed saliva samples were centrifuged at 2000 g for 10 min, yielding low-viscosity saliva. Salivary free cortisol concentrations were determined using a commercial chemiluminescence immunoassay with high sensitivity of 0.16 ng/ml (LIA, IBL Hamburg, Germany). Inter- and intra-assay variation was below 11.5 and 7.7%, respectively. All samples of one subject were analyzed in the same run.

### Psychological measures

#### Chronic stress

We assessed the amount of chronic stress over the last 3 months both at baseline and follow-up and by means of the Trier Inventory for the Assessment of Chronic Stress (TICS) [36]. The TICS is a 57-item questionnaire measuring 9 interrelated factors of chronic stress in addition to a screening scale for chronic stress (SSCS): 1) Work Overload, 2) Social Overload, 3) Pressure to Perform, 4) Work Discontent, 5) Excessive Demands at Work, 6) Lack of Social Recognition, 7) Social Tensions, 8) Social Isolation, and 9) Chronic Worrying. Using a 5-point rating scale ranging from 0 (never) to 4 (very often), participants were asked to rate how often they have had a certain experience or have experienced a certain situation within the last three months. Higher scores mean higher extent of chronic stress. Cronbach’s alpha was .87 in our sample for the SSCS indicating good reliability.

#### Control variables

We assessed contraceptive use, age at onset of sexual intercourse, total number of sexual partners, and smoking status and intensity based on previous findings suggesting associations with risk of genital HPV infection [37]. Smoking intensity was assessed by the average number of monthly smoked cigarettes. The total number of sexual partners was assessed by asking the participants how many sexual partners they have had in the past. Hormonal contraceptives were binary coded as using hormonal contraceptives vs. not using contraceptives. Moreover, we assessed sleep quality using the Pittsburgh Sleep Quality Index (PSQI) [38] due to its potential effects on cortisol responses [39, 40].

### Statistical analyses

Data were analyzed using SPSS Inc. version 23.0 for Mac OS X (Chicago, IL, USA) and presented as mean ± SEM. G*Power 3.1 defined an optimal sample size of *n* = 90 to detect a medium effect size of f = .30 in general linear models given two groups and the cortisol awakening response (CAR) or the daily area under the curve with respect to the ground (AUCgday [41]), respectively, as dependent variable with a power of 0.80. All tests were two-tailed with the significance level set at *p* ≤ .05 and the level of a statistical trend set at *p* ≤ .10. Data were tested for normal distribution using a Kolmogorov–Smirnov test before further statistical procedures were applied. We applied Huynh-Feldt correction for repeated measures. The CAR was calculated as the difference between the peak cortisol response (i.e., 30 min after awakening) minus cortisol level at awakening [42]. To evaluate total cortisol output over the study period, the AUCgday was calculated integrating the respective 0 min, 30 min, and 60 min after awakening, 11:00, 14:00, 17:00, and 20:00 cortisol sample. Body mass index (BMI) was calculated as the ratio of weight in kilograms to height in square meters. Across the study groups univariate analyses of variance (ANOVAs) were calculated to test for differences in group characteristics and chronic stress. To test for associations between chronic stress and HR-HPV status, logistic regression analyses were performed. To predict HR-HPV status at T1 or T2, we entered in a first step (model 1) potential HR-HPV risk factors (covariates) and in a second step (model 2) the chronic stress factor.

To examine the associations between cortisol and HR-HPV status, repeated measures ANCOVAs were applied with HR-HPV status as the group variable and the 3 (CAR) or 7 (AUCgday) time points at which cortisol was measured as repeated measures. To additionally test for group differences in the CAR and in the AUCgday, and single cortisol time points, we performed univariate ANCOVAs. To examine whether the cortisol measures (CAR, AUCgday) were mediators of the hypothesized associations between chronic stress and HR-HPV status, we performed mediator analyses using the PROCESS macro [43]. In the proposed mediation model chronic stress was tested as predictor and the HR-HPV status as outcome with the cortisol measures (i.e., CAR, AUCgday) as mediators. Statistical mediation of an association between predictor and outcome holds if the predictor is significantly associated with the mediator, and the mediator is significantly associated with the outcome while controlling for the predictor [44]. By default, the PROCESS macro uses a bootstrapping approach with 1000 bootstrap resamples and a 95% confidence interval of the indirect effects, which were adopted in this study.

In terms of covariates, we controlled a priori for the potential HPV risk factors contraceptive use, age at onset of sexual intercourse, number of sexual contacts, and smoking intensity [37] in all statistical analyses involving HR-HPV status at baseline and follow-up. Additionally, we have taken sleep quality into account as covariate in all statistical analyses involving cortisol measures. Effect size parameters (*f*) were calculated from partial *η*^*2*^-values and are reported where appropriate (effect size conventions: *f*: .10 = small, .25 = medium, .40 = large).

## Results

### Participant characteristics

Table 1 (left) provides the characteristics of participants per HR-HPV status at T1 (baseline). The HR-HPV positive group (*n* = 48) had more sexual partners during their lifetime and included more smokers than the HR-HPV negative control group (*n* = 140). No significant group differences were found in terms of age, BMI, employment, marital status, use of hormonal contraceptives, age at onset of sexual intercourse, sleep quality and numbers of cigarettes smoked per month. When comparing the HR-HPV positive group with the HR-HPV negative cortisol-control group (*n* = 41) the results did not significantly change, with the exception of age and numbers of cigarettes smoked per month. The HR-HPV positive group was older and engaged in heavier cigarette smoking when compared to the HR-HPV negative cortisol-control group. At T2 (follow-up), the HR-HPV positive group (*n* = 16) included less smokers and single women than the HR-HPV negative group (*n* = 26). No group differences were found in terms of age, BMI, employment, use of hormonal contraceptives, age at onset of sexual intercourse, number of cigarettes smoked per month, and sleep quality; see Table 1 [right]). See Table 2 for participants’ psychological characteristics per HR-HPV status at T1 and T2.

**Table 1:** Participants’ characteristics per HR-HPV status at baseline and at follow-up

	HR-HPV at baseline			*P*-value		HR-HPV at follow-up		*P*-value
	No (*n* = 140; control group; 1)	No (*n* = 41; cortisol-control group; 2)	Yes (*n* = 48; 3)	1 vs. 3	2 vs. 3	No (*n* = 26)	Yes (*n* = 16)	
Age (years), M ± SEM	24.9 ± .26	23.8 ± .44	25.1 ± .44	.63	.043	26.4 ± .62	26.2 ± .71	.81
BMI (kg/m^2^), M ± SEM	21.8 ± .25	21.2 ± .37	21.6 ± .51	.73	.55	21.6 ± .63	21.0 ± .51	.50
Employment, *n* (%)				.77	.25			.17
Yes	105 (75%)	27 (65.9%)	37 (77.1%)			23 (88.5%)	16 (100%)	
No	35 (25%)	14 (34.1%)	11 (22.9%)			3 (11.5%)	0	
Hormonal contraceptive use, *n* (%)	87 (62.2%)	26 (63%)	32 (67%)	.58	.75	17 (65%)	10 (62%)	.85
Age at onset of sexual intercourse, M ± SEM	17.1 ± .17	17.2 ± .33	16.6 ± .27	.13	.15	16.3 ± .35	16.8 ± .46	.42
Marital status, *n* (%)				.86	.90			.063
Single	38 (27.1%)	7 (17.1%)	18 (37.5%)			8 (30.8%)	2 (12.5%)	
Relationship	95 (67.9%)	34 (82.9%)	26 (54.2%)			18 (69.2%)	12 (75%)	
Married	7 (5%)	0	3 (6.2%)			0	2 (12.5%)	
Widowed	0	0	1 (2.1%)			0	0	
Total number of sexual partners, M ± SEM	7.1 ± 7.4	5.5 ± 5.6	10.8 ± 8.4	.005	.001	13.9 ± 13.1	8.9 ± 6.0	.16
Smoker, *n* (%)	43 (30.7%)	15 (36.6%)	25 (52.1%)	.008	.143	17 (65.4%)	5 (31.3%)	.075
Cigarettes per month, M ± SEM	106.4 ± 152.7	64.7 ± 116.2	128.1 ± 111.5	.54	.095	128.8 ± 125.1	99.2 ± 77.6	.63
Sleep quality (PSQI), M ± SEM	9.55 ± 1.93	9.39 ± 2.21	9.96 ± 2.13	.23	.23	4.77 ± 2.77	4.25 ± 1.69	.50

Notes. *BMI* body mass index, *PSQI* Pittsburgh Sleep Quality Index, global PSQI score (min. Score: 0, max. Score: 21)

**Table 2:** Participants’ psychological characteristics per HR-HPV status at baseline and at follow-up given as means and standard errors

	HR-HPV at baseline (T1)			*P*-value		HR-HPV at follow-up (T2)		*P*-value
	No (*n* = 140; control group)	No (*n* = 41; cortisol-control group)	Yes (*n* = 48)	1 vs. 3	2 vs. 3	No (*n* = 26)	Yes (*n* = 16)	
*TICS*								
Work overload	21.4 ± 0.50	21.2 ± 1.08	22.5 ± 0.94	.30	.37	23.4 ± 1.56	23.9 ± 1.82	.82
Social overload	14.1 ± 0.36	13.0 ± 0.68	14.1 ± 4.9	.99	.30	16.1 ± 1.06	15.8 ± 1.08	.84
Pressure to perform	24.6 ± 0.46	23.0 ± 0.86	24.1 ± 0.82	.59	.34	26.2 ± 1.07	24.4 ± 1.32	.32
Work discontent	18.2 ± 0.42	17.3 ± 0.80	19.1 ± 0.75	.29	.11	19.0 ± 1.00	19.4 ± 1.01	.78
Excessive demands at work	11.8 ± 0.28	10.9 ± 0.55	13.1 ± 0.66	.044	.017	12.7 ± 0.88	12.2 ± 1.05	.74
Lack of social recognition	8.7 ± 0.30	8.1 ± 0.5	8.4 ± 0.41	.53	.67	9.1 ± 0.62	9.3 ± 0.63	.83
Social tensions	11.4 ± 0.30	10.8 ± 0.48	10.7 ± 0.48	.29	.97	11.3 ± 0.74	13.1 ± 1.12	.18
Social isolation	12.7 ± 0.40	12.0 ± 0.82	13.1 ± 0.72	.68	.35	12.6 ± 1.00	13.3 ± 1.42	.69
Chronic worrying	10.7 ± 0.28	9.7 ± 0.57	11.4 ± 0.57	.29	.042	10.7 ± 0.82	12.3 ± 1.10	.25
Screening scale (SSCS)	28.8 ± 0.59	26.8 ± 1.35	30.3 ± 1.26	.21	.061	30.2 ± 1.99	32.1 ± 2.17	.54

### Associations between chronic stress and HR-HPV status

We calculated logistic regression analyses to address whether chronic stress was associated with the likelihood of being HR-HPV positive at T1 and T2. At baseline, higher levels of excessive demands at work were significantly related to HR-HPV positivity (b = .143, *p* = .022, OR = 1.15, 95% CI: 1.02–1.30), independent of contraceptive use, age at onset of sexual intercourse, number of sexual contacts, and smoking intensity. Moreover, higher levels of chronic worrying (b = .152, *p* = .032, OR = 1.16, 95% CI: 1.01–1.34) at T1 were related to HR-HPV positivity at T1, independent of contraceptive use, age at onset of sexual intercourse, number of sexual contacts, and smoking intensity. At follow-up, chronic stress levels remained the same (except for a slight increase in lack of social recognition, *p* = .025), but no significant associations with the likelihood of being HR-HPV positive emerged.

### Associations between cortisol and HR-HPV status

**Fig. 2:**
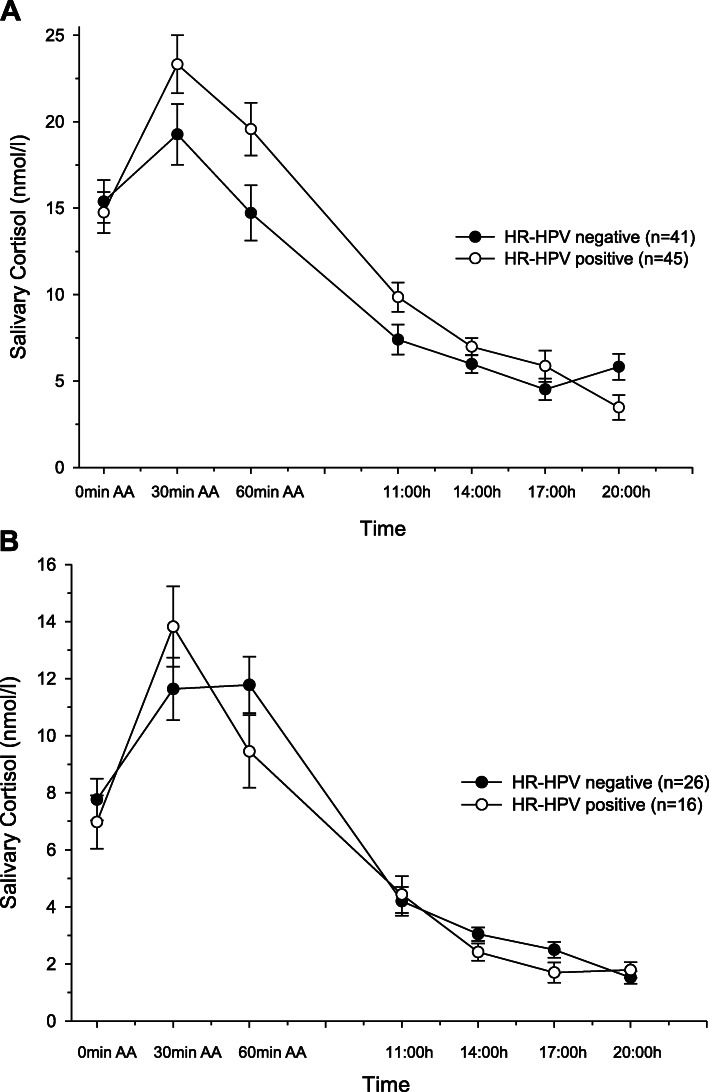
**a** & **b** Cortisol awakening response and cortisol output over the day in women with and without HR-HPV given as means and standard errors. AA, after awakening. **a**: At baseline. **b**: At follow-up

At baseline, the study groups significantly differed in post-awakening cortisol levels (interaction group-by-time: F (1.9/ 144.1) = 3.53, *p* = .035, f = 0.21; see Fig. 2a), independent of contraceptive use, age at onset of sexual intercourse, number of sexual contacts, smoking intensity, and sleep quality. Post hoc tests revealed no significant group differences in cortisol levels at awakening, but a significantly higher cortisol peak response to awakening in the HR-HPV positive group compared to the HR-HPV negative group (F (1/77) = 6.31, *p* = .014, f = 0.29). The groups also significantly differed in cortisol output over the day (interaction group-by-time: F (3.0/233.7) = 3.41, *p* = .018, f = 0.21), with a higher (integrated) overall cortisol secretion in the HR-HPV positive group as compared to the HR-HPV negative group by trend (AUCgday: F (1/77) = 3.714, *p* = .058, f = 0.22).

At follow-up, the study groups significantly differed in post-awakening cortisol levels (interaction group-by-time: F (2/70) = 3.93, *p* = .024, f = 0.34; see Fig. 2b), independent of contraceptive use, age at onset of sexual intercourse, number of sexual contacts, smoking intensity, and sleep quality. Post hoc tests revealed no significant group differences in cortisol level at awakening. However, on a trend level, the HR-HPV positive group showed a higher cortisol peak response to awakening than the HR-HPV negative group (F (1/35) = 3.71), *p* = .062, f = 0.33). The cortisol output over the day did not differ significantly between the HR-HPV positive group and the HR-HPV negative group (interaction effect group-by-time: *p* = .09; AUCgday: *p* = .50).

### Mediation of stress chronic stress effects on HR-HPV status by cortisol

Neither the CAR nor the AUCgday were found to mediate the associations between chronic stress (i.e., excessive demands at work and chronic worrying) and HR-HPV status at T1.

## Discussion

To the best of our knowledge, this is the first study that investigated the effects of chronic stress and different aspects of diurnal cortisol secretion on HR-HPV presence and persistence in young women.

As hypothesized, we found higher chronic stress levels, as measured by the TICS subscales excessive demands at work and chronic worrying to be associated with increased likelihood of HR-HPV positivity at baseline. These results were independent of potential HPV infection risk factors that were assessed at each measurement time point, including number of sexual contacts, age at onset of sexual intercourse, smoking intensity, and contraceptive use.

Our results provide first indications that chronic stress is an independent risk factor for HR-HPV presence in young women. This finding is in line with the hitherto single published human study that investigated associations between HPV infection and psychosocial stress. In this large-scale case-control study, bereaved women (i.e., loss of a family member due to death) showed a 62% increased risk of HR-HPV prevalence [25], but analyses were not adjusted for potential HPV infection risk factors. Thus, the independent effect of bereavement remains unclear. In light of the substantially increased risk of CIN grade 2 or worse diagnosis among women younger than 30 years with HR-HPV infections that persisted at least 12 months [4], our findings may suggest that in particular young women with chronic exposure to stressful demands are susceptible to HPV-associated CIN and cervical cancer. This assumption is strengthened by previous studies that have demonstrated a link between different kind of stressors, including life stressors [29, 31], intimate partner violence [28], passive/helplessness reactions to stress [27], parental death during childhood [26], bereavement [25], and the long-term consequences of persistent HR-HPV infections, i.e., cervical dysplasia and cancer. Notably, the groups did not differ in chronic stress levels at follow-up, which is most likely due to the reduction in the sample size (i.e., from *N* = 188 to *n* = 42). Future studies with longer follow-up periods are needed to verify the proposed role of chronic stress in HPV-associated cervical carcinogenesis.

Regarding mechanisms underlying the link between chronic stress and HR-HPV presence and persistence, our study is the first to investigate different aspects of diurnal cortisol secretion (i.e., CAR and AUCgday) as potential mediators for the association between stress and HR-HPV infection. As hypothesized, we found an increased cortisol awakening response and total cortisol output over the day in HR-HPV positive women compared to HR-HPV negative women at baseline. Likewise, at 12-months follow up, HR-HPV positive women also showed a slightly increased cortisol awakening response compared to HR-HPV negative women. Notably, the groups did not differ in their total cortisol output, which, again, is most likely due to the reduced sample size at 12-months follow up. Group differences were of medium to large effect size and independent of potential HPV infection risk factors. These findings provide first evidence for HPA axis hyperactivity in HR-HPV positive women. Considering the suppressive effects of increased cortisol levels on cell-mediated immune responses [21] that are important in HR-HPV clearance [6, 45] our results also support the suggestion of an active role for cortisol in the control of HR-HPV infection.

Interestingly, however, we were unable to confirm either cortisol awakening response or total cortisol output as mediators of the associations found between chronic stress and HR-HPV presence. For explanation of the missing mediation effect of cortisol awakening response regarding the chronic stress-HR-HPV relationship, we suggest three hypotheses. First, chronic stress and the CAR/diurnal cortisol output were measured on a different time scale (past 3 months vs. present day), which may explain the lack of association. Further studies employing long-term measures of HPA axis functioning (e.g., hair cortisol) may be more likely to uncover significant relationships. Second, the interaction between chronic stress, endocrine, and immune mechanisms in the field of HR-HPV infection might be too complex to be adequately covered by linear statistical models. Finally, it is possible that we were unable to detect a mediation effect of cortisol on the chronic stress-HR-HPV associations because our study was underpowered to reveal small to medium effects.

Our results may have clinical implications in that they suggest that stress management programs of young HR-HPV positive women with HPV axis hyperactivity and chronic exposure to stress could provide a psychobiological benefit. If stress management programs are directed at helping HR-HPV positive women to adaptively cope with demands of daily life, daily cortisol secretion and the course of HR-HPV infection might be positively affected. For example, it has previously been demonstrated that a stress management program using a combination of educative and cognitive-behavioral skills led to lower chronic stress and cortisol awakening response in participants suffering from chronic stress due to overwork [46].

The present study has several strengths, including the prospective study design that allowed us to investigate the role of chronic stress and cortisol in the course of HR-HPV infection. Moreover, HR-HPV test results were given to the participants after they had completed the study questionnaires. This is important to exclude confounding effects of distress due to being tested HR-HPV positive [47, 48] on the stress measures in our study. Furthermore, we controlled for a variety of potential HR-HPV risk factors to rule out confounding influence on the investigated associations. The study also has some limitations. It was an observational study, so that causal interpretations of the observed associations remain speculative. The generalizability of our findings might be limited to young women. Furthermore, because of the relatively small sample size and the follow-up period restricted to 12 months our data should be interpreted with caution until confirmed in larger studies with longer follow-up periods. Additionally, only the initially HR-HPV positive individuals were investigated at follow-up. It is possible that women with a negative HR-HPV status at baseline became infected at follow-up. Moreover, condom use has not been assessed in this study, and it cannot be ruled out that our cases with persistent infections may, in fact, have been cases with re-infections. Finally, the reduction of sample size for cortisol assessment in the HR-HPV negative group due to limited financial and organizational reasons must be mentioned as limitation.

## Conclusions

Taken together, we found first evidence that chronic stress and a dysregulated diurnal cortisol secretion might be independent risk factors for HR-HPV presence in young women. Future research is needed to replicate our findings in larger populations with longer follow-up periods.

## Data Availability

The dataset generated and analysed during the current study are not publicly available but are available from the corresponding author on reasonable request.

## Abbreviations

ANCOVA: Analyses of covariance
ANOVA: Analysis of variance
AUCg: Area under the curve with respect to ground
BMI: Body mass index
CAR: Cortisol awakening response
CIN: Cervical intraepithelial neoplasia
CMI: Cell-mediated immunity
CTL: Cytotoxic T lymphocyte
CVs: Inter- and intra coefficients of variation
GCs: Glucocorticoids
HPA: Hypothalamus-pituitary-adrenal
HR-HPV: High-risk human papillomavirus
PSQI: Pittsburgh Sleep Quality Index
SSCS: Screening scale for chronic stress
Th1: Type 1 T-helper
TICS: Trier Inventory for the Assessment of Chronic Stress
